# Estimating Bounds on Collisional Relaxation Rates of Spin-Polarized ^87^Rb Atoms at Ultracold Temperatures

**DOI:** 10.6028/jres.101.052

**Published:** 1996

**Authors:** Frederick H. Mies, Carl J. Williams, Paul S. Julienne, Morris Krauss

**Affiliations:** National Institute of Standards and Technology, Gaithersburg, MD 20899-0001

**Keywords:** *ab initio* calculations, cold trapped atoms, rubidium atom collisions, second-order spin-orbit, spin-relaxation rate, spin-spin interactions

## Abstract

We present quantum scattering calculations for the collisional relaxation rate coefficient of spin-polarized ^87^Rb(*f* = 2,*m* = 2) atoms, which determines the loss rate of cold Rb atoms from a magnetic trap. Unlike the lighter alkali atoms, spin-polarized ^87^Rb atoms can undergo dipolar relaxation due to both the normal spin-spin dipole interaction and a second-order spin-orbit interaction with distant electronic states of the dimer. We present *ab initio* calculations for the second-order spin-orbit terms for both Rb_2_ and Cs_2_. The corrections lead to a *reduction* in the relaxation rate for ^87^Rb. Our primary concern is to analyze the sensitivity of the ^87^Rb trap loss to the uncertainties in the ground state molecular potentials. Since the scattering length for the *a*^3^Σ^+^_u_ state is already known, the major uncertainties are associated with the *X*^1^Σ^+^_g_ potential. After testing the effect of systematically modifying the short-range form of the molecular potentials over a reasonable range, and introducing our best estimate of the second-order spin-orbit interaction, we estimate that in the low temperature limit the rate coefficient for loss of Rb atoms from the *f* = 2,*m* = 2 state is between 0.4 × 10^−15^ cm^3^/s and 2.4 × 10^−15^ cm^3^/s (where this number counts two atoms lost per collision). In a pure condensate the rate coefficient would be reduced by 1/2.

## 1. Introduction

The recent observation of Bose-Einstein condensation (BEC) in magnetically trapped alkali atoms [1,2,3] has brought to completion a 15 year attempt to achieve BEC in a weakly interacting atomic system [4]. The success of BEC in both ^87^Rb [1] and ^23^Na [3] and evidence for BEC in ^7^Li [2] were remarkable achievements brought about by the development of laser cooling during the past decade, the design of optical and magnetic traps for holding cold atomic samples, and most recently the development of evaporative cooling techniques to cool atoms below the recoil limit. This experimental success has renewed the interest in collisional loss rates for spin aligned alkali systems, since the binary and ternary collisional rates will limit the lifetimes of the experimental condensates. Experimentally, ^87^Rb is trapped in the (*f*_a_ = 2, *m*_a_ = 2) state, designated the doubly polarized state or the stretched state, for which binary hyperfine changing collisions could be the dominant loss process. Here *f*_a_ and *m*_a_ designate the quantum numbers for the total angular momentum of the ^87^Rb atom and its projection on some convenient space-fixed axis. On the other hand, ^23^Na atoms have so far been trapped in the (*f*_a_ = 1, *m*_a_ = − 1) state, which is theoretically expected to be more resistant to binary collisional loss. Hence, the lifetime of the ^23^Na condensate will probably be limited by three-body rates, although a condensate of stretched state ^23^Na atoms may be affected by binary collisional loss as well.

The purpose of this paper is to provide the most accurate calculations possible of the binary collision rates for all inelastic hyperfine scattering processes which can contribute to the loss of spin-polarized ground state ^87^Rb atoms at temperatures (*T* < 1 μK) associated with the recent experimental observation [1] of Bose-Einstein Condensation (BEC). This spin-relaxation is due to the following processes:

$$\begin{array}{c} {\;}^{87}\mathrm{Rb}(5s,f_{a}=2,m_{a}=2){+}^{\;87}\mathrm{Rb}(5s,f_{b}=2,m_{b}=2)\\ \to^{87}\mathrm{Rb}(5s,f_{a^{\prime }},m_{a^{\prime }}){+}^{\;87}\mathrm{Rb}(5s,f_{b^{\prime }},m_{b^{\prime }}). \end{array}$$ (1)

Having all ^87^Rb atoms in the stretched state with *m*_a_ = *f*_a_ is ideal for Bose-Einstein condensation, since inelastic collisions between such stretched states have very small rate coefficients. The entrance channel in Eq. (1) has a spin ***f*** = ***f***_a_ + ***f***_b_ of magnitude *f* = 4. Since the ^87^Rb atom has nuclear spin 3/2, this entrance channel can only project onto the triplet *a*^3^Σ^+^_u_ state of the atom pair which has total electron spin *S* = 1. Consequently a simple spin-exchange model of the collision [5,6] shows that stretched states do not relax during the collision. Our concern is determining the small but significant rate coefficient for the trap loss processes indicated in Eq. (1) that occur when the degeneracy of the molecule-fixed projection |*Ω*| = 0 or 1 of the *S* = 1 triplet potential is broken by relativistic forces. This splitting leads to spin-relaxation. We use standard quantum scattering methods to calculate the spin-relaxation event rate coefficient *K*_event_, defined by Stoof et al. [7], summed over all *f*_a_*_′_*,*m*_a_*_′_*,*f*_b_*_′_*,*m*_b_*_′_* channels that lead to loss of trapped atoms, namely channels for which either *f*_a_*_′_* and/or *f*_b_*_′_* ≠ 2. Such collisions lead to loss of both atoms from the trap because of the large kinetic energy release (equal to one or two units of ground state hyperfine splitting shared equally between the atoms). The total rate of spin relaxation in a Maxwellian gas (number of atoms lost per unit volume per unit time) is −2*K*_event_*n*^2^, where *n* is the density of *f*_a_ = 2,*m*_a_ = 2 atoms, since two atoms are lost per event represented by Eq. (1). If a condensate is present *K*_event_ is multiplied by $(2-{\bar{\xi }}^{2})/2$, where $\bar{\xi }$ is the condensate fraction [8].

Two atomic parameters are required to describe the separated atoms: the isotopic mass *m*_87_ = 158425.8*m*_e_ (where *m*_e_ is the electron mass = 9.109 × 10^−31^ kg) and the 6834.683 MHz splitting between the *f*_a_ = 2 and *f*_a_ = 1 hyperfine components. Assuming that the molecular hyperfine Hamiltonian can be adequately represented by a unitary frame transformation of the asymptotic atomic hyperfine Hamiltonians, the accuracy of the loss rate is basically limited by the accuracy of the molecular interactions we incorporate in our close-coupled scattering codes. To perform the dynamic calculations, we require four accurate molecular potentials *V*_S,_*_Ω_* (R): one defined by the ground *X*^1^Σ ^+^_g_ state with *S* = 0 and with molecule-fixed spin projection *Ω* = 0; and three defined by the lowest *a*^3^Σ ^+^_u_ state with *S* = 1 and spin projections *Ω* = 0, ± 1. These potentials take the following asymptotic form [9,10,11],

$$V_{0,0}(R)\sim -C_{\mathrm{exc}}e^{-aR}-(C_{6}R^{-6}+C_{8}R^{-8}+C_{10}R^{-10})$$ (2a)

$$\begin{array}{c} V_{1,0}(R)\sim +C_{\mathrm{exc}}e^{-aR}-(C_{6}R^{-6}+C_{8}R^{-8}+C_{10}R^{-10})\\ +{V^{\mathrm{SS}}}_{\Omega =0}(R)+{V^{\mathrm{SO}}}_{\Omega =0}(R) \end{array}$$ (2b)

$$\begin{array}{c} V_{1,\pm 1}(R)\sim +C_{\mathrm{exc}}e^{-aR}-(C_{6}R^{-6}+C_{8}R^{-8}+C_{10}R^{-10})\\ +{V^{\mathrm{SS}}}_{\Omega =\pm 1}(R)+{V^{\mathrm{SO}}}_{\Omega =\pm 1}(R) \end{array}$$ (2c)

where *V*^SS^*_Ω_*_=0_(*R*) = *α*^2^*R*^−3^ and *V*^SS^*_Ω=±_*_1_(*R*) = −1/2*α*^2^*R*^−3^ (*α* ≈ 1/137 is the fine structure constant) are the familiar spin-spin dipole terms that are primarily responsible for dipolar relaxation in hydrogen and the lighter alkalis. These spin-spin dipole terms have been elegantly treated in a series of papers by Verhaar and collaborators in Eindhoven [7,12]. The second-order spin-orbit terms *V*^SO^*_Ω_* which we have included are less well known in atomic collision physics, although they are well recognized as significant terms in molecular spectroscopy [13,14]. These terms, which are induced by spin-orbit interactions mediated through distant electronic states, mimic the effect of the direct spin-spin terms by introducing a splitting in the *Ω* = 0, ± 1 projections of the *S* = 1 state and can significantly modify the spin-relaxation rates. These terms will be discussed in detail in Sec. 5, where we will show that they are of opposite sign to the *α*^2^*R*^−3^ terms and tend to *diminish* the spin relaxation rate for ^87^Rb.

Our goal in this paper is to systematically assess the uncertainties in the spin-relaxation rates that are introduced by various uncertainties in the molecular parameters that enter into Eq. (2), and provide realistic bounds on the possible range of the loss rate coefficient *K*_event_ that can be expected for Rb_2_. All our rates are calculated using molecular potentials obtained from *ab initio* MCSCF codes employing the highly accurate *ab initio* pseudopotentials of Krauss and Stevens [10] for *R* < 20 *a*_o_ (1 *a*_o_ = 0.0529177 nm), and joined on to the well-analyzed long range dispersion potentials for the diatoms [11] for *R* > 20 *a*_o._ Because of the usual limitations of pseudopotentials and the typical convergence properties of *ab initio* calculations combined with the extraordinary demands we make on the required accuracy of the molecular potentials at ultra-cold collision energies, these potentials can only serve as excellent *initial* estimates of the short range portions of the *V*_S,_*_Ω_* (*R*) potentials in Eq. (2).

Collaborative work combining photoassociative spectroscopic data from the Texas group with theoretical analysis by the Eindhoven group [15,16] has done an excellent job of characterizing the *a*^3^Σ_u_^+^ potential, which controls the entrance channel dynamics for the spin relaxation described by Eq. (1). In Sec. 3, we examine the sensitivity of the scattering length to variations in this potential. We will accept the analysis of the Texas/Eindhoven group and have insured that our potential reproduces both the scattering length *A*_1_ ≈ + 110 *a*_o_ for the ^87^Rb isotope and *A*_1_ ≈ − 300 *a*_o_ for the ^85^Rb isotope. In addition, we introduce a useful new way to associate the scattering length with the binding energy of the last bound state in an attractive molecular potential.

In Sec. 4, we demonstrate a strong sensitivity of the relaxation rate to the shape of the *X*^1^Σ_g_^+^ singlet potential, when the *a*^3^Σ_u_^+^ scattering length is kept fixed at its known value. In this case the sensitivity is *not* due to the *a*^3^Σ_u_^+^ entrance channel, but depends on a *final-state* close coupling effect in the exit channels. The sensitivity to the potential leads to a factor of six uncertainty in the rate coefficient, which is analyzed using generalized MQDT theory [17] and especially the associated half collision amplitude version [18] of the theory. We also describe the interplay between the spin-spin (SS) and second-order spin-orbit (SO) contributions to the relaxation rate.

We previously had speculated that the SO terms would modify Rb spin-relaxation [19]. In Sec. 5 we present new *ab initio* calculations of these SO terms for Rb_2_ and Cs_2_. We also give our current understanding of the uncertainty range of the spin-relaxation rate of ^87^Rb. Finally, a summary of our results is presented in Sec. 6.

## 2. Scattering Theory of Ground State Alkali Atoms

In a field-free collision both the total angular momentum ***F*** = ***f***_a_ + ***f***_b_ + ***ℓ*** = ***f*** + ***ℓ*** and the total parity *p* = ± 1 are constants of the motion. The parity is the symmetry associated with the inversion of *all* electron and nuclear space-fixed coordinates through the center-of-mass of the dimer. The collision loss rate coefficient *K*_event_(*E*) for total collision energy *E* is expressed in terms of sums over *F* and *p* involving the scattering matrices ***S***(***F***,*p*,*E*) [8]. Each *S*-matrix is derived from a multichannel wave-function calculated from standard close-coupled codes for a given ***F***,*p*, and *E*, using an expansion in a channel state basis |*F*,*M*,*p*;*ℓ*,*f*,*f*_a_,*f*_b_〉, which describes the asymptotic properties of the separated atoms,

$$\Psi_{\gamma =\ell ff_{a}f_{b}}^{+F,M{,}_{p}}(E,R)=\sum_{\gamma^{\prime }=\ell^{\prime }ff_{a}^{\prime }f_{b}^{\prime }}|F,M,p;\gamma^{\prime }\rangle F_{\gamma^{\prime }\gamma }^{+}(E,R),$$ (3)

where *ℓ* is the angular momentum (partial wave) quantum number of the interatomic coordinate ***R***, *f* represents the magnitude of the channel angular momentum ***f*** = ***f***_a_ + ***f***_b_, and *γ* gives the spin channel in which the collision starts. The + indicates normal scattering boundary conditions for an incoming state in channel *γ* and outgoing spherical waves in channels *γ′*. The channel states are symmetrized with respect to interchange of the identical nuclei. One consequence of this symmetrization is that odd partial waves are missing from Eq. (3) for a collision of two *f*_a_ = 2, *m*_a_ = 2 ^87^Rb atoms.

In the absence of spin-spin and second-order spin-orbit interactions, the molecular Hamiltonian of two colliding ground state alkali atoms in (ns) orbitals, which can be expected to have zero total electronic *orbital* angular momentum ***L*** = (***ℓ***_a_ + ***ℓ***_b_) = 0, possesses two additional *almost* good quantum numbers. These are *ℓ* and *f*. Although this is not true when one or more of the atoms possesses electronic *orbital* angular momentum, such as an alkali in its first excited (*np*) orbit, it is an excellent approximation for the two colliding Rb(5*s*) atoms in Eq. (1). The physical reason is that for ***L*** = 0 there are no electrostatic interactions that cause locking of the electron spin angular momentum of the system to the internuclear axis. Ultimately, weak spin-spin dipole (SS) and second-order spin-orbit (SO) interactions cause the total electronic spin ***S*** = (***s***_a_ + ***s***_b_) to couple to the axis and lead to the small energetic splittings between the molecule-fixed spin projections *Ω* represented in Eq. (2). For the moment, if we ignore these latter interactions the *Ω* projections are perfectly degenerate and we can easily transform the asymptotic channel states |*F*,*M*,*p*;*ℓ*,*f*,*f*_a_,*f*_b_〉 into a basis defined by the total electron spin angular momentum *S* (***S*** = ***s***_a_ + ***s***_b_) and the total nuclear spin *I* (***I*** = ***i***_a_ + ***i***_b_), where ***s***_a_ and ***s***_b_ are the atomic electron spin angular momenta and ***i***_a_ and ***i***_b_ are the nuclear spin angular momenta (for ^87^Rb, *s*_a_ = *s*_b_ = 1/2 and *i*_a_ = *i*_b_ = 3/2). This transformation is

$$\begin{array}{l} |F,M,p;\ell ,f,S,I\rangle \propto \sum_{f_{a}f_{b}}\sqrt{\left(2S+1)(2I+1)(2f_{a}+1)(2f_{b}+1\right)}\\ \left\{\begin{array}{ccc} s_{a} & i_{a} & f_{a}\\ s_{b} & i_{b} & f_{b}\\ S & I & f \end{array}\right\}|F,M,p;\ell ,f_{a}f_{b}\rangle . \end{array}$$

In this new representation two adiabatic Born-Oppenheimer potentials, uniquely identified by the quantum numbers *S* = 0 (i.e., the *X*^1^Σ ^+^_g_ state) and *S* = 1 (i.e., the *a*^3^Σ ^+^_u_ state), appear on the diagonal of the Hamiltonian matrix. In the absence of SS and SO interactions the block of *S* channel states and the block of *I* channel states are diagonal and only couplings involving a simultaneous change in *I* and *S* are introduced by the hyperfine interactions. These coupling are constrained to sub-blocks which insure that ***f*** = ***I*** + ***S*** is conserved, and we find both *f* and *ℓ* remain perfectly good quantum numbers at all distances. Of course, at small distances the exchange splitting between the molecular potentials is large compared to the hyperfine splittings and hyperfine coupling is negligible. However, as we shall see, at distances of the order *R* ≈ 20 *a*_o_ to 40 *a*_o_, the hyperfine interaction becomes important and the *I* ↔ *S* coupling drives the system back into the asymptotically diagonal basis of channel states |*F*,*M*,*p*;*ℓ*,*f*,*f*_a_,*f*_b_〉.

As seen in Eq. (2), both SS and SO interactions produce an energy splitting of the *Ω* states which implies a locking of ***S*** to the internuclear axis. This effect is highlighted by applying a frame transformation of the |*F*,*M*,*p*;*ℓ*,*f*,*S*,*I*〉 channel basis such that *Ω* becomes represented as a “good” quantum number. However, in this new basis *f* and consequently *ℓ* are no longer conserved. Fortunately the splitting of the degeneracy of the $a^{3}\Sigma_{u}^{+}$ state is small, and hence *f* and *ℓ* still remain good approximate quantum numbers. This allows us to block the Hamiltonian for a given *F* and *p* into subspaces of common *f* and *ℓ* values. The atomic stretched state angular momenta, *f*_a_ = *f*_b_ = 2 and *m*_a_ = *m*_b_ = 2, can only couple to an *f* = 4 state, which then couples with the *ℓ* = 0 *s*-wave to give a total angular momentum *F* = 4. This is the only *F* value for which there is a stretched state *s*-wave. The spin-spin dipole interaction can only change *ℓ* by two units, and thus upon examining the *F* = 4, *p* = + 1 Hamiltonian, we find that the (*f* = 4, *ℓ* = 0) subspace can only couple directly to the (*f* = 3, *ℓ* = 2) and (*f* = 2, *ℓ* = 2) subspaces.

Our calculations are carried out with the full compliment of accessible channels associated with a given *F*,*M*,*p* and our rates are obtained with a sufficient summation over *F*,*M*,*p* to insure convergence. However, we find that at the temperatures relevant to BEC (*T* < 100 μK) only the single set of *F* = 4, *p* = + 1 solutions, which involve the close coupling of 20 channels in Eq. (3), contribute significantly to the stretched state spin relaxation. This is because only the incident *s*-wave contributes to spin relaxation, since the contributions from incident channels with *ℓ* ≥ 2 are strongly suppressed at these temperatures due to quantum threshold effects. Furthermore, of the 20 channels contributing to Eq. (3) for *F* = 4, *p* = + 1, and coupled by the 20 × 20 interaction matrix *U_γ,γ′_*(*R*), only the five *diabatic* channels labelled *γ* = 1–5 in Table 1 and consisting of the three subspaces described above, play any significant role.

Figure 1a shows the diagonal interaction potentials *U_γ,γ_* (*R*) for these five channels. Because of complicated curve crossings and strong interactions in the *γ* basis, more insight comes when we examine the five *α* = 1,5 “adiabatic” potentials *V_α_* (*R*) shown in Figs. 1a and 1b. These are obtained by diagonalizing the 5 × 5 interaction potential *U_γ,γ′_*(*R*) at each *R*. The five potentials never cross and are labeled in order of increasing energy. Each adiabatic channel *α* correlates asymptotically with the corresponding *γ* state in Table 1. At short *R* each *α* potential corresponds to a very good approximation with either the *X*^1^Σ^+^_g_ or *a*^3^Σ ^+^_u_ potentials. Thus, at short *R*, and out to where the exchange interaction term *C*_exc_ in Eq. (2) remains dominant, the *V_α_*_=1_(*R*) potential essentially mimics the pure *X*^1^Σ ^+^_g_ potential, and the other four *V_α_*(*R*) are basically pure *a*^3^Σ ^+^_u_ potentials.

Solving the set of five close-coupled equations and obtaining the 5 × 5 scattering matrix *S*(*γ*,*γ′*) = *S*(*α*,*α′*) for this abbreviated set of channels is sufficient to quantitatively reproduce the elaborate multichannel calculations to within a few percent at all energies below 100 μK. What has been defined [8] as the “event” rate constant *K*_event_ is simply the sum over all inelastic events experienced by the incident stretched state channel *γ = α* = 4,

$$K_{\text{event}}=\left(\frac{\pi \hbar }{\mu k}\right)\left[{\left|S_{1,4}\right|}^{2}+{\left|S_{2,4}\right|}^{2}+{\left|S_{3,4}\right|}^{2}+{\left|S_{5,4}\right|}^{2}\right]$$ (4)

where *μ* is the reduced mass of the Rb_2_ dimer, and *k* = √(2*μ****ϵ***/*ℏ*^2^) is the wave number of the *α* = 4 channel incident with relative kinetic energy ***ϵ***. At threshold the first three terms ${\left|S_{\alpha ,4}\right|}^{2}$ *α* = 1,2,3 vary as *k* and their contribution to *K*_event_ approaches a *constant* at low energies. These inelastic elements measure the coupling to the exothermic channels and invariably cause loss of *two* Rb atoms from the trap. The fourth term produces disorientation of the stretched-state atoms which ultimately leads to loss from the trap as well. However, as this element vanishes as ${\left|S_{5,4}\right|}^{2}$ ∝ *k*^2^, its contribution is negligible as *k*→ 0 and can be neglected.

The close coupled equations can be numerically solved using *either* the *α* or *γ* basis. Since these basis sets are asymptotically *equivalent*, they lead to exactly the same *S*-matrix. In actual practice the *γ* basis is vastly more convenient in solving the close-coupling, while the *α* basis is much more useful in gaining physical insight from the results. For example, examining the non-adiabatic coupling [17] we find that only channels *α* = 1 and *α* = 2 are strongly coupled, and this occurs over a very limited region *R* ≈ 20 *a*_o_ to 25 *a*_o_ (see below). All the remaining couplings are weak and perturbative. This latter feature will play an important role in assessing the sensitivity of the rates to the singlet potential in Sec. 4.

## 3. Sensitivity Analysis of the Triplet Scattering Length

All our rates are calculated using molecular potentials obtained from *ab initio* MCSCF codes employing the highly accurate *ab initio* pseudopotentials of Krauss and Stevens [10] for R < 20 *a*_o_, and joined on to the well-analyzed long range dispersion potentials for the diatoms [11] for R > 20 *a*_o._ Since the calculation of ultracold collision rates requires extraordinary accuracy of the molecular potentials, these potentials can only serve as excellent *initial* estimates of the short range portions of the *V_S,Ω_* (*R*) in Eq. (2). In particular, they are not accurate enough to confidently calculate threshold properties such as the scattering length. These parameters are determined by an integration of *V_S,Ω_* (*R*) over the entire range of *R*.

Since we have confidence in the long range parameters in Eq. (2), we only vary the short range potential in order to assess the sensitivity of the scattering calculations to these potentials. We have added an adjustable harmonic-like *short range* term to the *a*^3^Σ_u_^+^ and *X*^1^Σ_g_^+^ potentials as follows,

$${V^{\mathrm{sr}}}_{0,0}(R)=V_{0,0}(R)+C_{0}{\left(R-R_{e0}\right)}^{2}\;R<R_{e0}=7.89032a_{o}$$ (5a)

$${V^{\mathrm{sr}}}_{1,\Omega }(R)=V_{1,\Omega }(R)+C_{1}{\left(R-R_{e1}\right)}^{2}\;R<R_{e1}=11.6683a_{o}$$ (5b)

where Eq. (5) is only applied at distances *smaller* than the indicated equilibrium internuclear distances for the attractive singlet and triplet potentials respectively. Note that the functional form in Eq. (4) is chosen arbitrarily and has no physical significance; other short-range forms could be used with equal effect. In particular we want to show that by choosing an appropriate value for *C*_1_ in Eq. (4b) we insure that the associated *V*_1,_*_Ω_* (*R*) potentials give a scattering length of 110 *a*_0_ as suggested by the joint theoretical-experimental analysis of Boesten *et al*. [16].

**Fig. 2:**
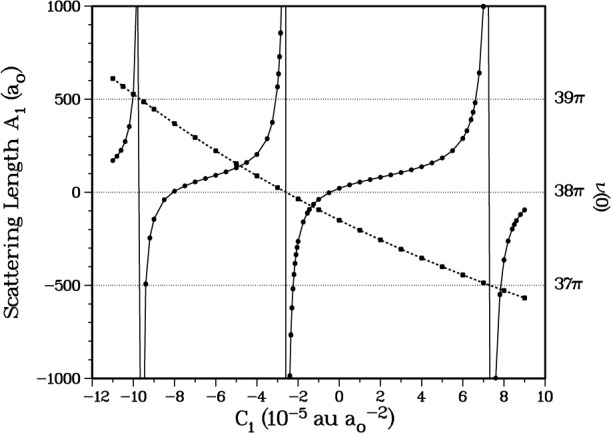
The variation of ^87^Rb_2_ scattering length *A*_1_ for the entrance *s*-wave channel incident on the *a*^3^Σ^+^_u_ potential, as a function of the short range *C*_1_ parameter, extracted from the wavefunction at *ϵ*/*k*_B_ = 0.1 nK (full circles). The solid line through the points gives the fit of Eq. (6). We also plot the threshold values *v*(0) of the bound state phase as we vary *C*_1_ (full squares). The modular π values of *v*(*ϵ_n_*) = *n*π identify the bound state eigenvalues. As the last bound state *ϵ_n_* eigenvalue approaches zero the predicted scattering length passes from a large *positive* value to a large *negative* value as the level “pops” out of the potential. This occurs at about *C*_1_= − 2.6 × 10^−5^ au/*a*_o_^2^ for ^87^Rb_2_ where *v*(0) → 38π.

Our initial fit to the *ab initio* calculations [10] for the *a*^3^Σ^+^_u_ potential happened to support 38 vibrational levels. The last level *v* = 37 has a binding energy of − 1.45618 × 10^−8^ au (1 au = *e*^2^/*a*_o_ = 4.359748 × 10^−18^ J, where *e* is the electron charge), and a positive scattering length of 21.6 *a*_o_. This corresponds to the point at *C*_1_ = 0 in Fig. 2 where we show the variation in scattering length as we systematically vary *C*_1_ in Eq. (5b). The figure shows that the scattering length *A*_1_ is extremely sensitive to the short-range portion of the potential. The scattering length is defined by the *s*-wave threshold behaviour of the elastic scattering phase shift *ξ*_1_ →−*kA*_1_ in the limit that the asymptotic wavenumber *k* goes to zero (*ℏ*^2^*k*^2^/2*μ* = ***ϵ*** with ***ϵ*** the collision energy). It is well known [20] that the actual value and the sign of *A*_1_ is critically dependent on the position of the *last bound state* that can be supported by a given potential. This in turn is related to what we like to call [17,21] the bound state phase *v*(***κ***), which is defined for negative energies *ϵ* =−*ℏ*^2^*κ*^2^/2*μ* which lie below the threshold at ***ϵ*** = 0, and where ***κ*** in turn is defined as a continuous *positive real* variable. The modular-π value of the bound state phase *v*(*κ_n_*) = *n*π locates the position of the *n*th vibrational eigenvalue [21,25], such that *ϵ_n_* =−*ℏ*^2^*κ_n_*^2^/2*μ*. Actually the deviation of the threshold value of *v*(0) from modular-π is a useful measure of the last bound state position. This quantity plays a prominent role in many descriptions of threshold behaviour (see Stwalley [22], LeRoy and Bernstein [23] and the Eindhoven group [16]), where it has been denoted as *v*_D_ and is sometimes called the effective vibrational quantum number at the dissociation limit. For our initial fit with *C*_1_ = 0 we found *v*(0) = 37.699541π. This quantity increases or deceases monotonically as we systematically make the potential more or less attractive by varying *C*_1_ in Eq. (4b). At 9.75 *a*_o_, the zero energy turning point of the *a*^3^Σ^+^_u_ potential for *C*_1_ = 0, a value of *C*_1_ = ± 5 × 10^−5^ au/*a*_o_^2^ produces a ± 50 cm^−1^ change in the potential. For comparison, the triplet state potential is 204 cm^−1^ deep at its potential minimum *R*_e1_.

Figure 2 shows that the scattering length as a function of the *C*_1_ shift parameter passes from plus to minus infinity as the last bound state is pushed out of the potential. The position of the singularity is easily located by examining the threshold behaviour of the bound state phase *v*(0). We see that this quantity approaches 38π just as *C*_1_ approaches − 2.6 × 10^−5^ au/*a*_o_^2^ and *A*_1_ passes through infinity. In fact a nice analytic relationship exists between the scattering length *A* and *v*(0),

$$A=-{\frac{\partial v}{\partial \kappa }|}_{\kappa \to 0^{+}}\left[\cot\left(\frac{\pi }{s-2}\right)+\cot\;v(0)\right],$$ (6)

where $\in =-\frac{\hbar^{2}\kappa^{2}}{2\mu }$ and the derivative is defined by $v(\kappa )=v(0)+{\frac{\partial v}{\partial \kappa }|}_{\kappa \to 0^{+}}\kappa$. The parameter *s* in cot(π/(*s* − 2)) is defined by the leading asymptotic power law − *C_s_*/*R^s^* for the potential. Both the *a*^3^Σ^+^_u_ and the *X*^1^Σ^+^_g_ potential have as the leading term *C*_6_*R*^−6^ and we have cot(π/(*s* − 2)) = 1. (Instead of the pure *a*^3^Σ^+^_u_ potential we actually prefer to use the adiabatic potential designated as *α* = 4 in Table 1, in which case the lower order *α*^2^*R*^−3^ terms in Eq. (2b) and (2c) are *rigorously* removed by the diagonalization of the interaction matrix and do not contribute to the threshold behaviour of this *s*-wave channel). If we evaluate *v*(*ϵ*) at an eigenvalue *ϵ* = *ϵ_n_* then $v(0)\approx n\pi -\frac{\partial v}{\partial \kappa }\kappa_{n}=n\pi +\delta_{n}$. In the special case, as *ϵ_n_* → − 0 and the last bound state lies just below the dissociation limit, such that $\delta_{n}\approx -\frac{\partial v}{\partial \kappa }\kappa_{n}\ll 1$ and tan *δ_n_* → *δ_n_*, we obtain the usual perturbative expression (Ref. [20] p. 48) for the scattering length

$$A\approx -\frac{\partial v}{\partial \kappa }\left[1+\frac{1}{\delta_{n}}\right]\to -\frac{\partial v}{\partial \kappa }\frac{1}{\delta_{n}}\to \frac{1}{\kappa_{n}}.$$ (7)

Although the conventional derivation of this expression is limited to a potential with a single bound state, we find perfect agreement with this limiting behavior even for wells supporting many bound states.

Actually, if we have a situation where *v*(0) is not quite ready to support the *n*^th^ bound state, i.e., $v(0)=n\pi -{\bar{\delta }}_{n}$ such that ${\bar{\delta }}_{n}\equiv -\frac{\partial v}{\partial \kappa }({\bar{\kappa }}_{n})\ll 1$, then we can visualize a “pseudobound” state lying just *above* the dissociation limit with an “eigenvalue” ${\bar{\epsilon }}_{n}=+\hbar^{2}{\bar{\kappa }}_{n}^{2}/2\mu$. In the limit where tan $v(0)\to -{\bar{\delta }}_{n}$ we obtain an expression which complements Eq. (7), $A\to -\frac{1}{{\bar{\kappa }}_{n}}$, and predicts a *negative* scattering length whenever a pseudobound state lies just *above* the dissociation limit. This behavior is well substantiated, and quantitatively confirmed, by the results in Fig. 2.

Numerical studies show that the bound state phase does indeed vary as $v(\kappa )=v(0)+\frac{\partial v}{\partial \kappa }\kappa$ near threshold, and furthermore the quantity $\frac{\partial v}{\partial \kappa }$ required in Eq. (6) is basically an *asymptotic* property that only depends on the long-range potential, and is totally insensitive to variations in the short range potential, such as those introduced by the shift parameter *C*_1_. From these data we estimate that $\frac{\partial v}{\partial \kappa }\approx -78\;a_{o}$. Using this estimate we have plotted Eq. (6) as the solid curve in Fig. 2 and find perfect agreement over the entire (modular π) range of *v*(0) with the calculated points. Note that this expression also predicts the exact locations of the zeros in the scattering length, which are always located at *v*(0) = *n*π + 0.75π. It is interesting to note that over the modular p range the scattering length is predicted to be *positive* for 3/4 of the range, and *negative* for only 1/4. This means that, if we know nothing about the short range potential, we can at least predict *there is a 3:1 probability that the scattering length will be positive!* The functional form of Eq. (6) was also confirmed for an asymptotic *R*^−8^ by setting *C*_6_ = 0 in Eq. (2), such that cot(π/(*s* − 2)) = cot(π/6) = √3.

**Fig. 3:**
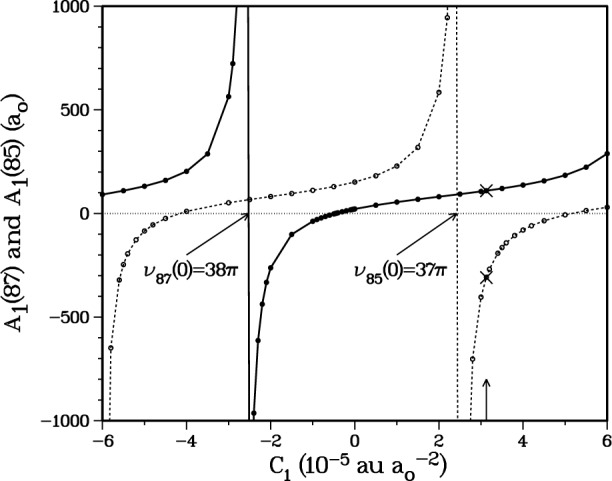
Scattering lengths as in Fig. 2, for the ^87^Rb(solid circles) and ^85^Rb(open circles) isotopes. These are evaluated for the same *a*^3^Σ^+^_u_ potential, using the appropriate mass for each isotope. For all future calculations we use *C*_1_ = 3.13 × 10^−5^ au/*a*_o_^2^, indicated by the vertical arrow. This choice yields scattering lengths *A*_1_(87) = 109.1 *a*_o_ and *A*_1_(85) = − 309.3 *a*_o_, as prescribed by the analysis given by the Texas/Eindhoven group [15,16].

If we choose *C*_1_ = 3.128 × 10^−5^ au/*a*_o_^2^ we obtain a scattering length *A*_1_(87) = + 109.1 *a*_o_ and a threshold value of *v*_87_(0) → 37.3761π, which predicts 38 bound vibrational levels with a last bound state at *E*_37_ = − 2.947925 × 10^−9^ au. This choice is made to conform to the scattering length obtained by the Texas/Eindhoven group [15,16]. Further confidence is obtained from Fig. 3 where we compare the scattering lengths for the ^87^Rb and the ^85^Rb isotopes. In calculating the two scattering lengths, the only difference is the mass of the two isotopes. We see that at the same value of *C*_1_ the calculated scattering length *A*_1_(85) = − 309.1 *a*_o_ for ^85^Rb is in good agreement with [15,16]. In addition, the threshold value *v*_85_(0) = 36.9367π predicts 37 vibrational levels with a last bound state at *E*_36_ = − 3.305245 × 10^−8^ au.

**Fig. 4:**
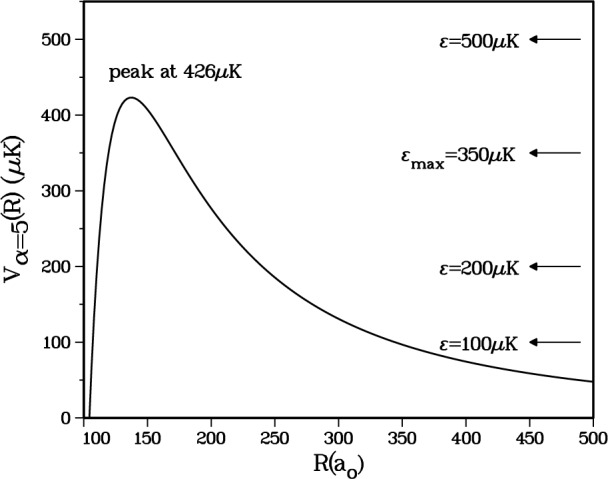
The *ℓ* = 2 centrifugal barrier for the *α* = 5 channel, which asymptotically correlates with the *a*^3^Σ^+^_u_ potential. The four energies indicated will be used in Fig. 5.

**Fig. 5:**
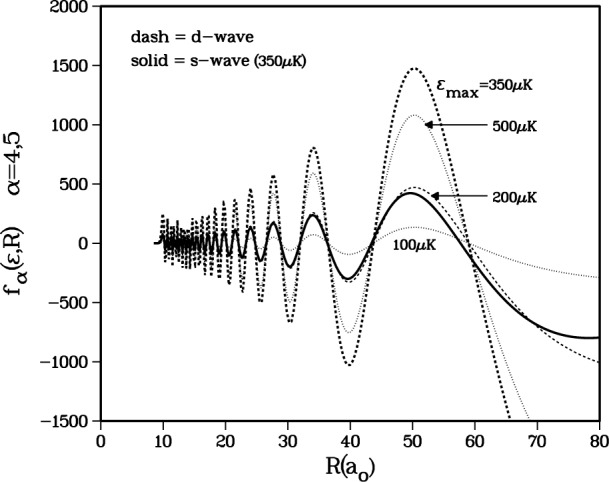
Energy normalized continuum wavefunctions for the adiabatic *α* = 5 channel for energies *ϵ* = 100 μK, 200 μK, 350 μK, and 500 μK (dashed lines) and for the adiabatic *α* = 4 channel for energy *ϵ* = 350 μK (solid line). The adiabatic *α* = 5 channel exhibits *d*-wave shape resonance structure with maximum amplitude enhancement near *ϵ*_max_ = 350 μK.

One final confirmation of the validity of the *a*^3^Σ_u_^+^ potential we are using is obtained by examining the *d*-wave shape resonance structure defined by the adiabatic channel *α* = 5 in Table 1. This corresponds to an incident channel which correlates with the triplet state at short distance, and correlates with the *f*_a_ = *f*_b_ = 2 atomic states at large distance entering with an *ℓ* = 2 partial wave. This gives rise to the centrifugal barrier shown in Fig. 4, with a barrier height of 420 μK. The radial wavefunctions *f_α_*_=4_(*R*) and *f_α_*_=5_(*R*) are shown in Fig. 5 for four incident kinetic energies: 100 μK, 200 μK, 350 μK, and 500 μK. These *f_α_* (*R*) are the single-channel, energy normalized elastic scattering wavefunction associated with the *V_α_* (*R*) adiabatic potential. Boesten et al. [16] have concluded that there is a shape resonance enhancement of the photoassociation from channel *α* = 5 relative to the *α* = 4 channel with incident *ℓ* = 0. The solid curve shows the *α* = 4 wavefunction for 350 μK. The *α* = 5 channel amplitude increases with energy up to *ϵ* ≈ 350 μK, after which it decreases. One would infer from these plots that the *d*-wave shape resonance is very broad with a “width” extending from 200 μK to 500 μK. This behaviour is consistent with the analysis given in reference [16].

## 4. Sensitivity of Loss Rates to the Singlet Potential

**Fig. 6:**
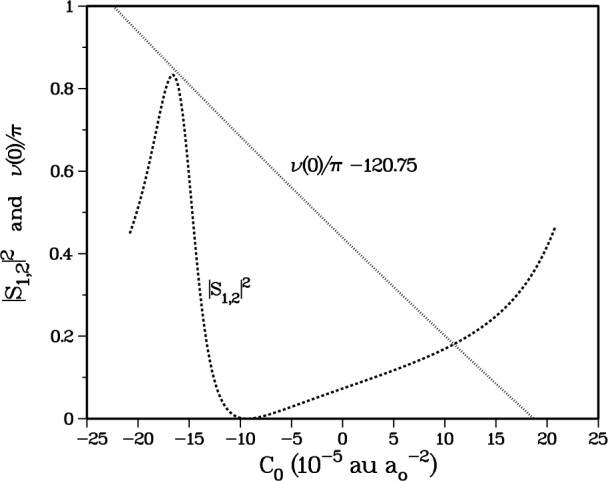
Variation of |*S*_12_|^2^ (for a total energy *ϵ*/*k*_B_ = 0.1 μK incident in channel 4) as a function of the short-range *C*_0_ parameter (dashed line) and variation of *v*(0) as a function of the same parameter (dotted line). At *C*_0_ = 8.0 × 10^−5^ au/*a*_o_^2^ the quantity *v*(0)/π = 121 and the *X*^1^Σ^+^_g_ potential supports exactly 122 vibrational states. At this point the singlet scattering length passes through infinity. However, |*S*_12_|^2^ varies smoothly through this region, because channels 1 and 2 have high asymptotic kinetic energy, well away from threshold.

As we systematically varied the short-range *X*^1^Σ^+^_g_ potential by varying *C*_0_ in Eq. (5) we find a surprisingly large, and seemingly erratic variation in the stretched state loss rate coefficient. The spin-relaxation rates are determined by the very small *S*(*α*,4) matrix elements, which typically have a magnitude of 10^−3^. We found that the variation was intimately associated with the variation of the large |*S*(*α*=1, *α*=2)|^2^ element shown in Fig. 6. As seen from Fig. 1 and Table 1, this *S*-matrix element measures the probability for the *f*_a_,*f*_b_ = 1,2 → *f*_a_,*f*_b_ = 1,1 transition. The coupling which determines this transition probability is the short-range exchange potential, which causes the strongest and only non-perturbative inelastic event associated with the channels in Table 1. Since *S*(1,2) is evaluated at a total energy determined by the *α* = 4 entrance channel, namely 0.1 μK above the *α* = 4 channel threshold, the asymptotic kinetic energies in channels *α* = 1 and *α* = 2 are 656 mK and 328 mK, respectively (see Table 1).

Before we can understand the strong influence of the *X*^1^Σ^+^_g_ potential on the perturbative *S*(*α*,4) elements we must first examine the profound effect of this potential on the behaviour of *S*(1,2). The change to the short range potential is sufficient to make small displacements in the nodes of *f_α_*_=1_(*R*) in the coupling region which is important for determining *S*(1,2), but it is not immediately obvious why this leads to such large variations in the above-threshold *S*(1,2). Fig. 6 shows |*S*(1,2)|^2^ varies from a minimum of 0 at *C*_0_ ≈ − 9.4 × 10^−5^ au/*a*_o_^2^ to a maximum of 0.834 at *C*_0_ ≈ − 16.4 × 10^−5^ au/*a*_o_^2^.

**Fig. 7:**
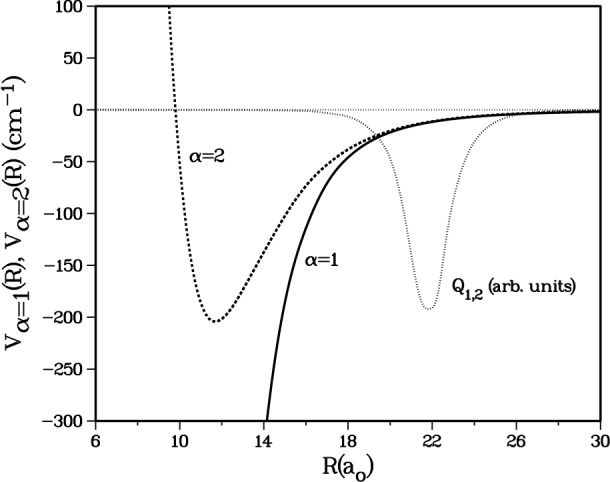
The adiabatic potentials for channels *α* = 1 and *α* = 2 together with the non-adiabatic coupling operator (in arbitrary units) between these channels. The latter is well localized in the region of 18 *a*_0_ < *R* < 26 *a*_0_, where the exchange splitting between the *X*^1^Σ^+^_g_ and *a*^3^Σ^+^_u_ potentials becomes comparable to the hyperfine splitting.

This variation is best understood by considering the matrix element of the radial coupling operator $Q_{1,2}(R)\frac{\partial }{\partial R}$ [17] between the adiabatic states *α* = 1,2 in Table 1 and Fig. 7. In the first order distorted wave approximation (Ref. [20] p. 349, and Refs. [26 and 27]) the *S*(1,2) matrix element is proportional to the integral,

$$S(1,2)\propto \int dRf_{1}(R)Q_{1,2}(R)\frac{\partial }{\partial R}f_{2}(R),$$

where *Q*_12_(*R*) is determined by the *R*-variation of the orthogonal 5 × 5 matrix *M*_ij_(*R*) which diagonalizes the diabatic interaction matrix, $U(R)=\tilde{M}(R)V(R)M(R)$, such that

$$Q_{12}(R)=\sum_{j=1,5}M_{1,j}(R)\partial M_{2,j}(R)/\partial R.$$

Not surprisingly we find the coupling is highly localized in the vicinity of *R* ≈ 22 (see Fig. 7) where the spin-exchange splitting between the singlet and triplet potential in Eq. (2) becomes comparable to the hyperfine splitting of the atoms. Although *Q*_12_ causes strong nonadiabatic mixing between channels 1 and 2, the distorted wave approximation above is suitable for the qualitative argument we make below, even though it is not suitable for quantitative calculations.

**Fig. 8:**
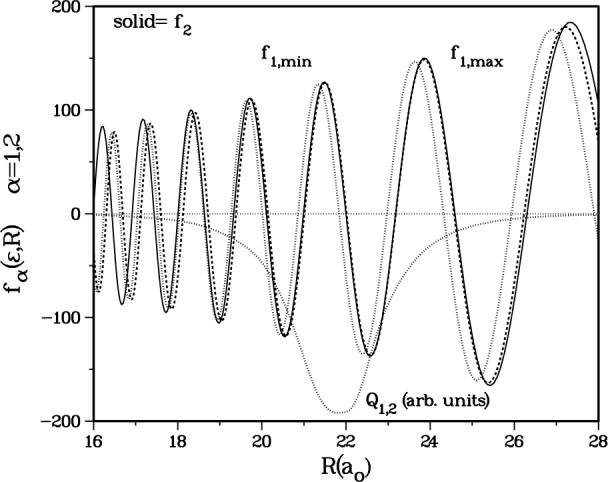
The adiabatic *f*_2_ wavefunction (solid curve) and the adiabatic wavefunctions *f*_1,max_ (dotted curve) and *f*_1,min_ (dashed curve). The latter were calculated using the singlet shift parameters *C*_0_ = − 16.4 × 10^−5^ au/*a* ^2^ o and *C*_0_ = − 9.80 × 10^−5^ au/*a*_o_^2^, which produce the maximum and the minimum inelastic |*S*_12_|^2^ elements in Fig. 7 respectively. The *Q*_12_ operator (in arbitrary units) is also shown, and locates the region of strong non-adiabatic coupling between channels 1 and 2.

Changing the singlet potential has a negligible effect on the adiabatic *f*_2_(*R*), which is shown by the solid curve in Fig. 8 and primarily portrays a pure triplet state, at least up to the vicinity of strong coupling near 22 *a*_o_. The two different short range singlet potentials, associated with the indicated minimum and maximum |*S*(1,2)|^2^ in Fig. 6, are used to obtain the two different *f*_1_ functions which we designate as *f*_1,min_ and *f*_1,max_ in Fig. 8. Note the structure of these functions in the vicinity of the peak of the non-adiabatic coupling operator *Q*_12_. The *f*_2,min_ function has an almost perfect overlap with the *f*_1_ function. Since the complete non-adiabatic operator is equal to *Q*_12_ times the radial derivative ∂/∂*R* this perfect overlap implies a very poor overlap between *f*_1_ and ∂*f*_2,min_(*R*)/∂*R*, and therefore implies that the distorted wave integral should be quite small. In fact, for this case our *exact* close-coupling results yield |*S*(1,2)|^2^ = 0.000017 (presumably by varying *C*_0_ slightly we could have found a perfect cancellation with |*S*(1,2)|^2^ ≡ 0). The second function *f*_2,max_ in Fig. 8 is phase shifted with respect to *f*_1_, implying improved overlap between *f*_1_ and ∂*f*_2,max_(*R*)/∂*R* and a larger distorted wave integral. For this case our close-coupling predicts the maximum |*S*(1,2)|^2^ = 0.83.

**Fig. 9:**
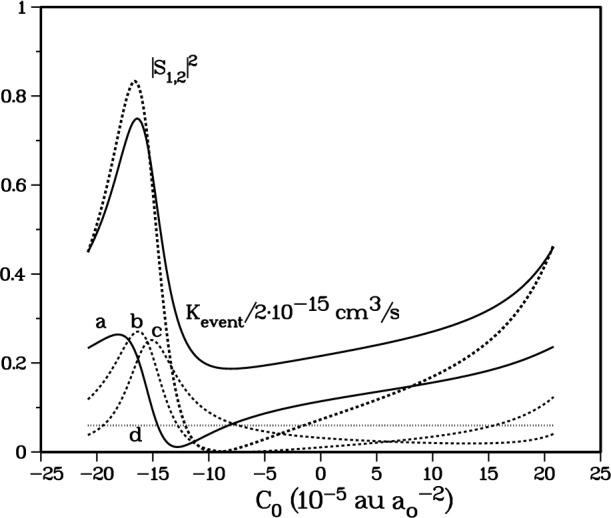
|*S*_12_|^2^ and *K*_event_ versus the short-range *C*_0_ parameter for the ground *X*^1^Σ^+^_g_ potential. The figure shows the strong correlation between the two quantities. The correlation with specific components *K*_event_(22,22 → *f*_a_*m*_a_, *f*_b_*m*_b_) are also shown: (a) *f*_a_*m*_a_, *f*_b_*m*_b_ = 11,11; (b) *f*_a_*m*_a_, *f*_b_*m*_b_ = 22,10; (c) *f*_a_*m*_a_, *f*_b_*m*_b_ = 21,11; (d) *f*_a_*m*_a_, *f*_b_*m*_b_ = 22,11. All rate coefficients have been divided by 2 × 10^−15^ cm^3^/s in order to place them on the same scale as the dimensionless |*S*_12_|^2^.

Our initial fit to the *ab initio* calculations [4] for the ^1^Σ^+^_g_ potential, combined with the ^3^Σ^+^_u_ potential with a scattering length *A*_1_ = 109 *a*_0_ prescribed by Ref. [16], just happened to yield a value of |*S*(1,2)|^2^ = 0.0731. When the ^1^Σ^+^_g_ potential was systematically modified at short distances by increasing *C*_0_, with nodes being systematically pushed to larger distances, Fig. 9 shows that *K*_event_ tracks the associated values of |*S*(1,2)|^2^ with *K*_event_ varying by roughly a factor of 4. The figure also shows that several of the individual components contributing to the spin-relaxation show similar qualitative variation with differences in detail.

**Fig. 10:**
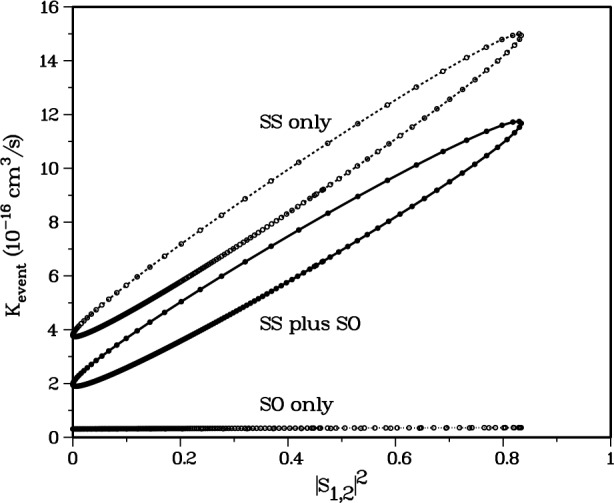
Rate coefficient *K*_event_ versus |*S*_12_|^2^ as *C*_0_ is varied for the *X*^1^Σ^+^_g_ potential. The variation of *C*_0_ is sufficient to “pop” two bound states out of the *X*^1^Σ^+^_g_ potential. Three such curves are shown. The curve labeled “SS only” includes the spin-spin (SS) interaction only. The lowest curve labeled “SO only” includes the second-order spin-orbit (SO) interaction only. The middle curve labeled “SS plus SO” includes both the SS and SO interactions.

Figure 10 shows the behavior of *K*_event_ with respect to the variation in ${\left|S(1,2)\right|}^{2}$. We show the dependence of the stretched state rate coefficients on the *X*^1^Σ^+^_g_ potential for three different situations. The *SS-only* curve gives *K*_event_ when we only include the usual spin-spin splitting which varies as 3*α*^2^/2*R*^3^. The *SO-only* curve shows the very small rate coefficient, almost independent of *S*(1,2), that results if the SS splitting is removed and *only* the short-range second-order spin-orbit splitting is included. In Sec. 5 we will show that the *SO-only* contribution primarily occurs at short distance, *R* < 20 *a*_o_, where significant molecular interactions can occur. Consequently, the dependence of the *SO-only* rate coefficient on |*S*(1,2)|^2^ is minuscule. Finally the *SS-plus-SO* curve shows the rate coefficient when both SS and SO interactions are included in the close-coupling calculation. This curve demonstrates up to a factor of two *reduction* in the rate coefficient when compared to the SS-curve

The rate coefficients in Fig. 10 are double valued as a function of |*S*(1,2)|^2^ except at the extremes of the range. This property can be traced to Fig. 9, where we have followed |*S*(1,2)|^2^ over a range of *C*_0_ that changes *v*(0)/π by unity and “pushes” one bound state out of the *X*^1^Σ^+^_g_ potential well. We find two identical values of |*S*(1,2)|^2^ in this interval. The second value occurs when the *f*_1_ function is shifted by *approximately half* a deBroglie wavelength in the peak region of *Q*_12_, thereby maintaining the same value of the distorted wave integral for |*S*(1,2)|^2^ (see Fig. 8). This changes the sign of *S*(1,2) but results in the same |*S*(1,2)|^2^. However, this change in sign results in slightly different *interference* effects in the evaluation of *S*(*α*,4) and thus *K*_event_ at distances beyond *R* ≈ 26 *a*_o_, as we will discuss below. Actually, in Fig. 10 we varied *C*_0_ enough to “eject” two bound states from the potential, and, for a given resultant |*S*(1,2)|^2^ the rate coefficients in Fig. 10 can not be distinguished.

At the extrema of *K*_event_ in Figs. 9 or 10, it is possible to associate a single *A*_0_ scattering length for the *X*^1^Σ^+^_g_ potential with the particular |*S*(1,2)|^2^ value. This is not possible away from the extrema, since there are two values that correspond to the same *K*_event_. The minimum in *K*_event_ corresponds to a scattering length for the *X*^1^Σ^+^_g_ potential of *A*_0_ = + 95 *a*_o_ and the maximum corresponds to a value of *A*_0_ = + 54 *a*_o_. If the scattering length were measured to be near one of these values, then the relaxation rate will be near one of its extreme values.

We will now present a qualitative argument why varying the magnitude of *S*(1,2) affects the magnitude of the spin-relaxation rate involving the *S*(*α*,4), *α* ≠ 4, matrix elements. Since the coupling is weak it is an excellent approximation to represent these elements as follows:

$$S(\alpha ,4)\propto \sum_{\alpha^{\prime }}\langle F_{\alpha^{\prime },\alpha }^{-}|Q_{\alpha^{\prime },4}(R)\frac{\partial }{\partial R}|f_{4}^{+}\rangle$$ (8)

where $F_{\alpha^{\prime }\alpha }^{-}$ are defined in the same manner as in Eq. (3), but now in the adiabatic channel basis and with an outgoing state in channel *α*. Each column vector in the matrix *F*^−^(*R*) defines the radial components of a five-channel close-coupled outgoing wavefunction for each of the adiabatic channels *α* = 1,5. The function $f_{4}^{+}$ represents the incoming wavefunction of the *α* = 4 adiabatic potential of Fig. 1. This can be viewed as a generalized, multichannel version of the Distorted Wave Approximation [20,26,27]. The *Q_α′_*_,4_ matrix elements introduce the weak spin-dependent coupling between adiabatic channels 4 and *α′*.

At short distances, to the left of the strong coupling region in Fig. 8 the solutions *F*^−^(*R*) are simply proportional to the 5 × 5 diagonal matrix of adiabatic reference functions *f_α_*

$$F^{-}(R)\propto f(R)e^{-i\xi }=\delta_{\alpha^{\prime },\alpha }f_{\alpha }(R)e^{-i\xi \alpha }\;\text{for}\;R<20\;a_{o}.$$ (9a)

Applying generalized MQDT theory [17] and its associated half collision amplitude [18] to the outgoing multichannel functions at distances beyond about *R* = 26 *a*_o_, the exact close-coupled wavefunctions can be represented rigorously as follows

$$F^{-}(R)\propto [f(R)+g(R)Y]{\left(1+iY\right)}^{-1}e^{-i\xi }\;\text{for}\;R>26\;a.u.$$ (9b)

where *g*(*R*) = *δ_α,α′_g_α_* (*R*) and *g_α_* (*R*) is an irregular solution for the adiabatic *V_α_* (*R*) potential. Alternatively, this may be written using running wave reference functions:

$$F^{-}=\frac{i}{2}[h^{+}-h^{-}\Sigma^{*}]e^{-i\xi },\;h^{\pm }=g\pm if\to k^{-1/2}e^{\pm i(kR+\xi )}$$ (9c)

where the real symmetric *Y* matrix is related to the close-coupled scattering matrix *S* = e*^iξ^*Σe*^iξ^* = e*^iξ^*(1 + *iY*) (1 − *iY*) ^−1^e*^iξ^*. The only *Y* matrix element of any magnitude in this ultracold five channel system is *Y*_1,2_; therefore, we need only consider the *α* = 1 and 2 channels and we can reduce *F*^−^ to a 2 × 2 matrix for these two channels. For the weak coupling case in Fig. 8 even the *Y*_1,2_ element is negligible and the structure of *F*^−^(*R*) remains diagonal as in Eq. (9a) for all *R*. However, if the chosen *X*^1^Σ^+^_g_ potential yields a large *S*(1,2), the structure in Eq. (9b) can strongly influence Eq. (8). It is not a bad approximation [17] to represent a 2 × 2 *Y* matrix as follows,

$$Y=\left[\begin{array}{cc} d & x\\ x & -d \end{array}\right]\approx \left[\begin{array}{cc} 0 & x\\ x & 0 \end{array}\right]$$ (10)

where, especially if one uses an adiabatic representation, the diagonal elements *d* are generally negligible. For the strong coupling case, |*S*(1,2)| = |Σ(1,2)| can have a maximum value approaching unity.

For the simple model used here, we use *x* = 1 for the strong coupling case and *x* = 0 for the weak coupling case. For these two extreme cases the function *F*^−^ in Eq. (9b) takes a simple form. For the two channels *α* = 1 and *α* = 2 the relevant matrix elements of *F*^−^ are:

$$F^{-}=\frac{1}{2i}\left[\begin{array}{ll} h_{1}^{+}e^{-i\xi_{1}} & ih_{1}^{-}e^{-i\xi_{2}}\\ ih_{2}^{-}e^{-i\xi_{1}} & h_{2}^{+}e^{-i\xi_{2}} \end{array}\right]\;x=1,\;|S(1,2)|=1$$ (11s)

$$F^{-}=\left[\begin{array}{cc} f_{1}e^{-i\xi_{1}} & 0\\ 0 & f_{2}e^{-i\xi_{2}} \end{array}\right]\;x=0,\;|S(1,2)|=0$$ (11w)

We designate these two cases “s” and “w” respectively, for strong and weak inelastic scattering probability measured by *S*(1,2). Using these limits in Eq. (8) we find

$$\begin{array}{l} S(1,4)\propto -\frac{i}{2}\langle h_{1}^{+}e^{-i\xi_{1}}|V_{1,4}|f_{4}\rangle +\frac{1}{2}\langle h_{2}^{-}e^{-i\xi_{1}}|V_{2,4}|f_{4}\rangle \\ S(2,4)\propto -\frac{i}{2}\langle h_{2}^{+}e^{-i\xi_{2}}|V_{2,4}|f_{4}\rangle +\frac{1}{2}\langle h_{1}^{-}e^{-i\xi_{2}}|V_{1,4}|f_{4}\rangle \end{array}$$ (12s)

or

$$\begin{array}{l} S(1,4)\propto \langle f_{1}e^{-i\xi_{1}}|V_{1,4}|f_{4}\rangle \\ S(2,4)\propto \langle f_{2}e^{-i\xi_{2}}|V_{2,4}|f_{4}\rangle \end{array}$$ (12w)

The radial functions *f*_1_ and *f*_2_ are just the elastic scattering standing waves shown in Fig. 8 and oscillate strongly against the standing wave *f*_4_ defined by the incident channel. Thus, we expect small values for the matrix elements in Eq. (12w). In the strong coupling regime the matrix elements involve the overlap of *f*_4_ with pure outgoing (or incoming) running waves with amplitudes which do not oscillate with *R*, and we can easily understand why the matrix elements in Eq. (12s) are much larger than those in Eq. (12w). The enhancement of the spin-relaxation rate coefficient in the presence of strong final state interactions is well demonstrated in Figs. 9 and 10.

**Fig. 11:**
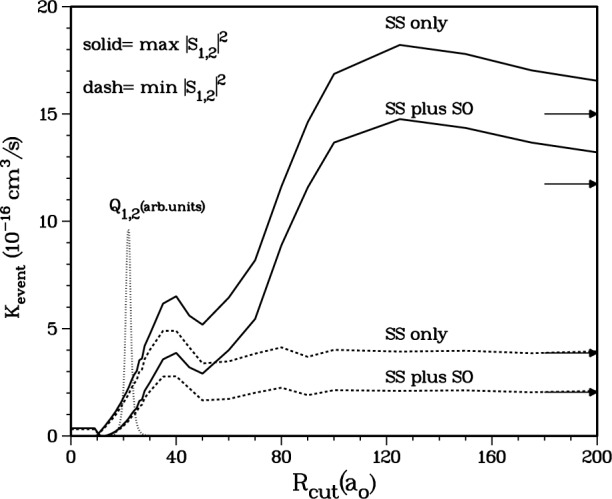
*K*_event_ versus the artificial cutoff parameter *R*_cutss_ of the spin-spin interaction. The *V*^SS^(*R*) coupling is set to zero for *R* > *R*_cut_. *K*_event_ is shown for the two *C*_0_ values which yield the largest (solid lines) and smallest (dashed lines) inelastic |*S*_12_|^2^. Two curves are shown for each choice of *C*_0_. One corresponds to including the SS interaction only, and the other corresponds to including both the SS and SO interactions (the SO coupling in not cut-off). The arrows show the values for *R*_cut_ → ∞.

Further insight into this final state effect can be gleaned by evaluating *K*_event_ while systematically limiting the range of the spin-spin coupling in Eq. (2). We do this by assuming the fine structure constant *α* has its usual constant value 1/137 up to some cutoff distance *R*_cut_ and then vanishes identically for *R* > *R*_cut_. The results of such a study are shown in Fig. 11. The solid curves show the result using the *X*^1^Σ^+^_g_ potential which yields the maximum |*S*(1,2)|^2^. In this case we expect Eqs. (10s) and (11s) to prevail at distances larger than the peak of the *Q*_12_ operator, which is shown by the dotted curve in Fig. 11. The dashed curves show the corresponding results for the minimum |*S*(1,2)|^2^ case, for which Eqs. (11w) and (12w) should be valid for all distances. There are two sets of curves. Each set has one line with the SS interaction only and one with our best estimate of the second-order spin-orbit terms included. Since these SO terms are short ranged and their principle contributions occur at distances to the left of the *Q*_12_ operator, we did not apply a cutoff to these terms.

Both dashed curves in Fig. 11, associated with the standing-wave weak coupling solutions in Eq. (11w), have achieved their asymptotic values by about *R* ≈ 40 *a*_o_–60 *a*_o_. Any possible contributions to the loss-rates beyond this region are quenched by standing wave oscillations in the matrix elements Eq. (12w). On the other hand, as shown by the behavior of the pair of solid curves, running wave terms in the strong field Eq. (12s) continue to add to the rates out to distance beyond *R* = 200 *a*_o_ and finally reach full convergence at about *R* ≈ 300 *a*_o_.

We now discuss the interesting effect that the total loss rates are decreased when we include the *V*^SO^ terms as well as the *V*^SS^ terms. The distorted wave approximation in Eq. (8) implies that the *S*(4,*α*) *S*-matrix elements can be separated into an SS and an SO term. The loss rate depends on the square of *S*-matrix elements:

$${\left|S^{\mathrm{SS}}+S^{\mathrm{SO}}\right|}^{2}={\left|S^{\mathrm{SS}}\right|}^{2}+{\left|S^{\mathrm{SO}}\right|}^{2}+S^{\mathrm{SS}}S^{\mathrm{SO}*}+S^{\mathrm{SS}*}S^{\mathrm{SO}}.$$

The first two terms sum the individual contributions to the rate, whereas the last terms exhibit the interference between them. Figures 10 and 11 show that |*S*^SO^|^2^ is independent of the strength of the exit channel coupling measured by |*S*(1,2)|^2^ and is always small compared to |*S*^SS^|^2^. On the other hand, |*S*^SS^|^2^ does depend very strongly on the strength of the exit channel coupling. Obviously the interference effect is more significant for the case of weak SS coupling than for the case of strong SS coupling. In the former case, *K*_event_ is decreased by a factor of 2 when SO coupling is included, whereas in the latter case, the decrease is only 20 %. Including the SO terms causes a decrease in *K*_event_, since the SO coupling has an opposite sign from the SS coupling, as discussed in the next section.

## 5. Evaluation of Second-Order Spin-Orbit Coupling

The doubly spin polarized atomic states on the left hand side of Eq. (1) can only be relaxed by the weak coupling between the two electron spins. Two terms, shown in Eq. (2), contribute to the effective spin-spin interaction Hamiltonian: the *direct* spin-spin dipole interaction *V*^SS^*_Ω_* (*R*), varying at long range as 1/*R*^3^, and the indirect second-order spin-orbit interaction *V*^SO^*_Ω_* (*R*). The latter originates when the atomic charge clouds overlap as a molecule is formed, and the interaction between the ground state spins are modified due to couplings mediated through distant excited electronic states of the molecule. These interactions are well known in molecular spectroscopy and mimic the direct spin-spin coupling for a ^3^Σ state [13,14]. The reason these interactions mimic spin-spin coupling is that they split the *Ω* = 0 and ± 1 components in a similar manner as the direct spin-spin terms. For heavy species like Rb and Cs we will see that these indirect terms can be much larger than the *V*^SS^*_Ω_* (*R*) term at short distances and strongly influence the spin-relaxation rate.

We have calculated *ab initio* molecular spin-orbit matrix elements to obtain estimates of the second-order spin-orbit correction terms *V*^SO^*_Ω_* (*R*) in Eq. (2). For the *a*^3^Σ_u_^+^ state these terms are mediated through distant electronic states of ^1^∏_u_, ^3^∏_u_, ^5^∏_u_, and ^3^Σ_u_^−^ symmetry. We calculate the *R*-dependent spin-orbit matrix elements for these states using all-electron wavefunctions for the molecular states generated by standard *ab initio* methods. The second-order interaction for the *a*^3^Σ_u_^+^ state is dominated by the matrix elements $\langle a^{3}\Sigma_{u,\Omega }^{+}{\left|H_{\mathrm{SO}}(R)\right|}^{2S+1}\Pi_{u,\Omega }\rangle$ involving ^1^∏_u_ and ^3^∏_u_ states which correlate to the first excited ^2^S + ^2^P atomic asymptotes. In this case the second-order coupling term due to a specific ^2S+1^∏_u,_*_Ω_* state takes the form [14]

$$V_{\Omega }^{\mathrm{SO}}(S,R)=b^{\mathrm{SO}}(S,\Omega )Ps(R)$$ (13a)

where

$$b^{\mathrm{SO}}(S,0)=0,\;b^{\mathrm{SO}}(S,\pm 1)=1\;\text{for}\;S=0$$ (13b)

$$b^{\mathrm{SO}}(S,0)=2,\;b^{\mathrm{SO}}(S,\pm 1)=1\;\text{for}\;S=1$$ (13c)

and

$$P_{s}(R)=-\frac{|\langle^{3}\Sigma_{u,\Omega =1}|H_{\mathrm{SO}}{|}^{2S+1}\Pi_{u,\Omega =1}\rangle {|}^{2}}{V{(}^{2S+1}\Pi_{u},R)-V{(}^{3}\Sigma_{u},R)}.$$ (13d)

Here *H*_SO_(*R*) is the electronic spin-orbit coupling operator and *Ω* = *Λ* + *Σ*, where *Λ* is the projection of the electronic orbital angular momentum and *Σ* is the projection of the electron spin angular momentum on the internuclear axis. Since the important aspect of the second-order spin orbit coupling for dipolar relaxation is the *splitting* it introduces between *Ω* = 0 and ± 1 components of the ^3^Σ_u_^+^ state, we represent the second-order spin-orbit couplings as an “effective” spin-spin coupling term $V_{\Omega =0}^{\mathrm{SO}}(R)$ as follows,

$$\begin{array}{l} V_{\Omega =0}^{\mathrm{SO}}(R)=\frac{2}{3}[P_{1}(R)-P_{0}(R)]\\ V_{\Omega =\pm 1}^{\mathrm{SO}}(R)=-\frac{1}{3}[P_{1}(R)-P_{0}(R)] \end{array}$$ (14)

Since *P*_S_(*R*) decays exponentially to large *R*, and its magnitude at short *R* is small compared to uncertainties in the short range ^3^Σ_u_^+^ potential, we can ignore the mean contribution of these interactions which equals [3*P*_1_(*R*) + *P*_0_(*R*)]/2 and assume that our adjustment of the ^3^Σ_u_^+^ potential to fit the experimental-theoretical estimate of the scattering length in Sec. 3 actually incorporates this mean spin-orbit contribution.

**Fig. 12:**
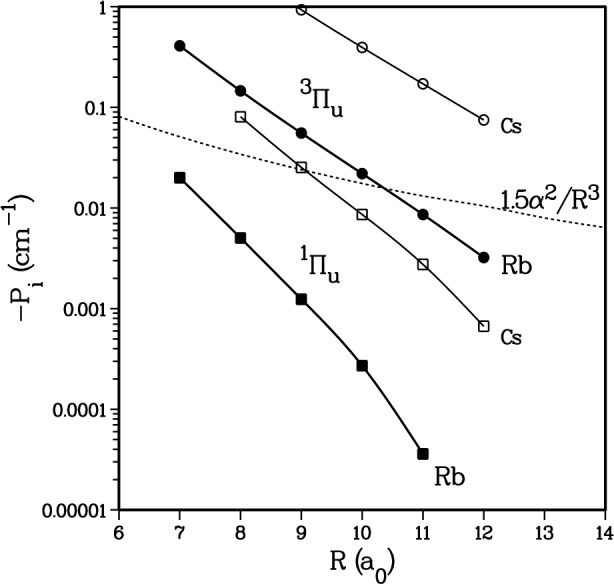
Calculated second-order spin-orbit coupling parameter of Eq. (11d) as a function of the internuclear separation *R*. The circles show the contribution from the first excited ^3^∏_u_ state, and the squares show that from the ^1^∏_u_ state. Solid points are for Rb_2_ and open ones for Cs_2_. The dashed line shows the difference between the *Ω* = 0 and |*Ω*| = 1 components of the spin-spin interaction.

Figure 12 shows our calculated *P*_0_(*R*) and *P*_1_(*R*) for Rb_2_ and Cs_2_. The b^3^∏_u_ state, which is energetically closest to the ^3^Σ_u_^+^ state, has the largest coupling, and *P*_1_ is an order of magnitude larger than *P*_0_. Figure 12 also shows that *P*_0_ and *P*_1_ have a much shorter range than the 1/*R*^3^ spin-spin term. To a good approximation we find we can fit the numerical *ab initio* results to the following expression,

$${V^{\mathrm{SO}}}_{\Omega =0}(R)=-2{V^{\mathrm{SO}}}_{\Omega =1}(R)=-C\alpha^{2}e^{-B(R-R_{S})}$$ (15)

where for Rb_2_ *C* = 0.001252 au, *B* = 0.975 *a*_o_^−1^, and for Cs_2_ *C* = 0.02249 au, *B* = 0.830 *a*_o_^−1^ and *R*_S_ = 10 *a*_o_ for both; here *C* is given in au $\left(1\;\mathrm{au}=\frac{e^{2}}{a_{o}}=4.359748\times 10^{-18}\;J\right)$. Note that $V_{\Omega =0}^{\mathrm{SO}}$ is *negative* compared to the *positive α*^2^/*R*^3^ spin dipolar term in Eq. (2b) and at about *R* ≈ 10.33 *a*_o_ these two terms exactly cancel in the case of Rb_2_. The same is true for Eq. (2c). Since it is the *difference* between the potentials from Eqs. (2b) and (2c) that gives rise to spin-relaxation, and the overall magnitude of these differences is reduced by the addition of the spin-orbit interaction, we might anticipate the spin-dipolar relaxation rates will be *reduced* accordingly. However, we should emphasis that although this is true for spin aligned Rb, where the two contributions are of similar magnitude, the opposite effect appears likely for Cs, since the magnitude of the second-order spin-orbit term for Cs_2_, although still negative, is much larger than the spin-spin term. The overall effect of the spin-orbit term in Cs_2_ is to *increase* the spin-relaxation rate relative to that predicted by spin-spin only.

**Fig. 13:**
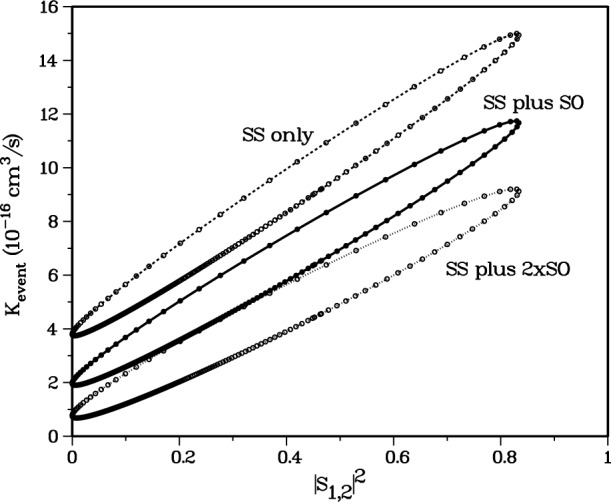
Rate coefficient *K*_event_ versus |*S*_12_|^2^ as C_0_ is varied for the *X*^1^Σ^+^_g_ potential. The upper two curves are the same as in Fig. 10. The three curves indicate the uncertainty in *K*_event_ due to the uncertainty in the *X*^1^Σ^+^_g_ potential and in the SO interaction. The three curves show three different cases of the strength of the second-order spin-orbit (SO) interaction: the upper curve has no SO interaction, the middle curve uses our calculated *ab initio* values, and the lower curve uses twice the calculated values.

To test the sensitivity of *K*_event_ to the magnitude of the spin-orbit coupling we have arbitrarily multiplied Eq. (15) by a factor of two and recalculated the loss-rates. Although it is impossible to place unequivocal error bounds on our calculations of $V_{\Omega }^{\mathrm{SO}}$, it is unlikely that our error in estimating *V*^SO^ is as much as a factor of two. Such a variation does give a reasonable bound to the possible effect of the coupling. In Fig. 13 we compare the rates for the SS-only calculation to those calculated using *V*^SO^ and 2 × *V*^SO^ added to *V*^SS^. This figure also summarizes the status of our current confidence in *K*_event_ for ^87^Rb. Since we are not able to determine the ^1^Σ^+^_g_ potential with sufficient accuracy to place any constraint on the magnitude of |*S*_12_|^2^, the rate coefficient is spanned by the range shown by the curve labeled “SS plus SO” in Fig. 13. The loss rate coefficient, 2*K*_event_, lies between 0.4 × 10^−15^ cm^3^/s and 2.4 × 10^−15^ cm^3^/s. If our calculated *V*^SO^ should be erroneous, we still expect the loss rate coefficient to lie between 0.1 × 10^−15^ cm^3^/s and 3.0 × 10^−15^ cm^3^/s. The lower bound is determined by the “SS plus 2 × SO” curve, and the upper bound by the “SS only” curve. It must also be remembered that this assessment is based on having a precise scattering length for the *a*^3^Σ^+^_u_ potential. If the estimate of *A*_1_ = 110 *a*_o_ supplied by the Texas/Eindhoven should be in error, then the rate coefficient will be modified accordingly.

We wish to make one final point before we conclude our discussion of the spin-order effects. Figure 12 shows that *V*^SO^ is about an order of magnitude larger for Cs_2_ than for Rb_2_. In fact, the contribution to spin-relaxation of doubly spin-polarized Cs atoms from the second-order spin-orbit term will dominate that due to the spin-spin term. Model calculations for Cs suggest that spin-relaxation rates for Cs are insensitive to the singlet potential. This agrees with our result for Rb shown by the nearly horizontal curve labeled “SO only” in Fig. 10. The spin-relaxation rate for Cs atoms is likely to be much larger than that for Rb atoms, but the actual value will still depend on the *a*^3^Σ^+^_u_ potential.

## 6. Conclusions: Assessment of Current Accuracy of Calculated Rb_2_ Loss Rates

In conclusion, we have determined the uncertainty in the spin-relaxation rate coefficient for stretched state ^87^Rb atoms associated with uncertainties in the molecular parameters which control the magnitude of the relaxation cross section. The stretched state relaxation affects the lifetime of the experimentally observed ^87^Rb condensate [1,24]. The condensate described in [1] remains dilute enough that its lifetime, ignoring collision with background thermal atoms, is determined by the binary stretched state loss rates [24]. Ternary rates will become dominant in more dense condensates.

Our calculations use the $a^{3}\Sigma_{u}^{+}$ or stretched state scattering length *A*_1_ = 110 *a*_0_ as required by the experimental photoassociation data for spin-polarized ^87^Rb atoms [16]. Our calculations show that the lack of experimental knowledge of the $X^{1}\Sigma_{g}^{+}$ potential provides the largest source of uncertainty in determining the spin-relaxation rate coefficient. The *s*-wave entrance channel for collision of the *f*_a_ = 2 + *f*_b_ = 2 stretched state atoms only couples very weakly to the two possible exit channels of the spin-relaxation process, which produce *f*_a_ = 1 + *f*_b_ = 2 or *f*_a_ = 1 + *f*_b_ = 1 separated atoms with increased kinetic energy. The uncertainty in spin-relaxation rate is associated with the strength of mixing between the two components in these exit channels. We varied the inner wall of the $X^{1}\Sigma_{g}^{+}$ potential in order to determine the range of uncertainty. Our calculations show that the loss rate coefficient due to spin-relaxation, 2*K*_event_, is uncertain to about a factor of 6, lying in the range 0.4 × 10^−15^ cm^3^/s to 2.4 × 10^−15^ cm^3^/s. In a pure condensate the rate coefficient is simply *K*_event_ [18]. Doubly polarized Rb may have the smallest collisional loss rate coefficient of any the alkali species if the rate coefficient lies near the lower end of its estimated range.

We have provided *ab initio* estimates for the second-order spin-orbit terms $V_{\Omega }^{\mathrm{SO}}(R)$ which contribute to the effective spin-spin interaction. For the heavier alkali atoms these terms have an important effect on the spin relaxation rate. In ^87^Rb, the $V_{\Omega }^{\mathrm{SO}}(R)$ terms causes a reduction in the rate coefficient for the collisional relaxation of stretched state atoms throughout the possible parameter space for varying the $X^{1}\Sigma_{g}^{+}$ potential. The decrease ranges between a factor of 2 % and 20 %, depending on whether the rate coefficient lies near the lower or higher end of its range of uncertainty. Our calculations suggest that the contribution of the $V_{\Omega }^{\mathrm{SO}}(R)$ terms to the spin-relaxation rate coefficient of stretched state Cs atoms will be much larger than that from the spin-spin dipole term.

Our analysis points out a critical need for more precise determinations of the $X^{1}\Sigma_{g}^{+}$ and $a^{3}\Sigma_{u}^{+}$ potentials for Rb_2_ and Cs_2_ and of the spin-coupling parameters. Experimental determination of $V_{\Omega }^{\mathrm{SO}}(R)$ could be approached by precision spectroscopy on the fine and hyperfine structure of the $a^{3}\Sigma_{u}^{+}$ state. The quantum chemistry community could provide more complete and accurate calculations of the second-order spin orbit interactions as a function of *R*.

## Figures and Tables

**Fig. 1a:**
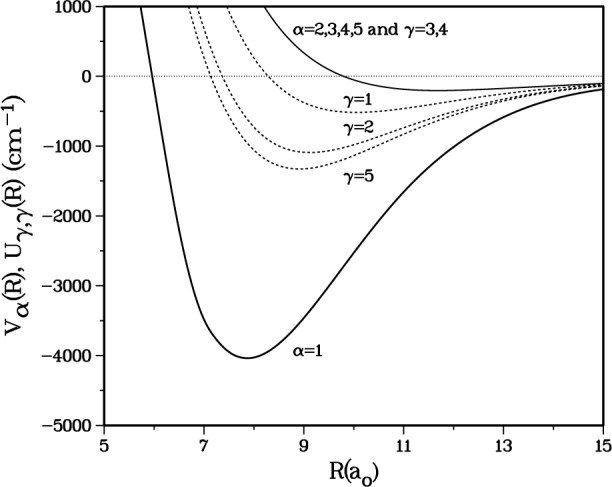
Diagonal potentials for the five most significant *F* = 4, *p* = + 1 channels. The dashed curves show diagonal elements of the *γ* = 1–5 case(e) diabatic interaction matrix *U_γ,γ_* (*R*). The solid curves show the adiabatic potentials *V_α_* (*R*) for *α* = 1–5 in Table 1. On the scale of this figure the two *γ* = 3,4 and the four *α* = 2,3,4,5 channels essentially track the *a*^3^Σ^+^_u_ potential. The *γ* = 1,2,5 potentials are admixtures of *V*_0,0_ and *V*_1,_*_Ω_* and are strongly coupled by off-diagonal terms proportional to the exchange term in Eq. (2). The adiabatic *α* = 1 channel tracks the *X*^1^Σ^+^_g_ potential.

**Fig. 1b:**
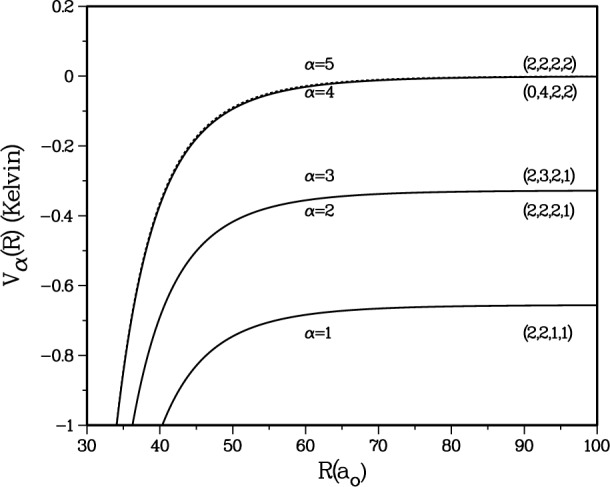
Asymptotic correlations of five adiabatic potentials *α* = 1–5 in Table 1.

**Table 1:** Significant *F* = 4, *p* = + 1 channel states for spin-depolarization rates near threshold

Diabatic (asymptotic) basis						Adiabatic Basis	
*γ*	*ℓ*	*f*	*f*_a_	*f*_b_	Asymptotic energy (mK)	*α*	Short *R* label
1	2	2	1	1	−656.022	1	^1^Σ^+^_g_
2	2	2	2	1	−328.011	2	^3^Σ^+^_u_
3	2	3	2	1	−328.011	3	^3^Σ^+^_u_
4	0	4	2	2	0.000	4	^3^Σ^+^_u_
5	2	2	2	2	0.000	5	^3^Σ^+^_u_
